# A Sponge-Driven Elastic Interface for Lithium Metal Anodes

**DOI:** 10.34133/2019/9129457

**Published:** 2019-09-15

**Authors:** Han Yu, Jian Xie, Na Shu, Fei Pan, Jianglin Ye, Xinyuan Wang, Hong Yuan, Yanwu Zhu

**Affiliations:** ^1^Hefei National Research Center for Physical Sciences at the Microscale and Department of Materials Science and Engineering and CAS Key Laboratory of Materials for Energy Conversion, University of Science and Technology of China, Hefei, Anhui 230026, China; ^2^iChEM (Collaborative Innovation Center of Chemistry for Energy Materials), University of Science and Technology of China, Hefei, Anhui 230026, China

## Abstract

The lithium (Li) metal is one promising anode for next generation high-energy-density batteries, but the large stress fluctuation and the nonuniform Li deposition upon cycling result in a highly unstable interface of the Li anode. Herein, a simple yet facile engineering of the elastic interface on the Li metal anodes is designed by inserting a melamine sponge between Li and the separator. Driven by the good elasticity of the sponge, the modified Li anode maintains a Coulombic efficiency of 98.8% for 60 cycles and is cyclable at 10 mA cm^−2^ for 250 cycles, both with a high capacity of 10 mA h cm^−2^. We demonstrate that the sponge can be used to replace the conventional polypropylene as a porous yet elastic separator, showing superior cycling and rate performance as well. In addition to the efficiency of the elastic interface on the cycling stability, which is further confirmed by an in situ compression-electrochemistry measurement, the porous structure and polar groups of the sponge demonstrate an ability of regulating the transport of Li ions, leading to a uniform deposition of Li and the suppression of Li dendrites in cycling.

## 1. Introduction

Although lithium ion batteries (LIBs) have attained a huge success over the past few decades, state-of-the-art anodes based on graphite are unable to meet the growing demands for energy storage capability. Due to the high specific capacity (3860 mA h g^−1^), low density (0.534 g cm^−3^), and low electrochemical potential (-3.04 V versus standard hydrogen electrode), metallic lithium (Li) has been considered as the optimal anode, especially in recently developed batteries such as Li-sulfur batteries and Li-air batteries [1]. Compared to graphite anodes that store Li ions via an intercalation reaction, the Li metal anode boosts the energy density with direct plating/stripping across the anode-electrolyte interface [2]. The high reactivity of the Li metal leads to an instantaneous formation of a heterogeneous and fragile solid electrolyte interphase (SEI) on the Li surface [3–5]. During Li plating, the dramatic volume expansion and nonuniform stress may bring cracks in SEI [6]. Subsequently, part of the Li metal comes into direct contact with the electrolyte, and the reactions continuously consume Li and the electrolyte, resulting in low Coulombic efficiency (CE) [7, 8]. In addition, the Li ions tend to deposit on the cracked SEI due to the lower energy barrier, causing the intensified inhomogeneous Li deposition [9] and the dendrite formation [10, 11]. In Li stripping, on the other hand, the dendrites may be broken, which expedites the formation of “dead Li” [3, 12]. The repeated formation/breaking of SEI and the accumulation of “dead Li” further deteriorate CE, and the dendrite growth also causes a short circuit and significant problems related to safety [3, 13].

Considerable efforts have been devoted to overcoming these challenges related to the unstable interface. One strategy is to modify the properties of the anode-electrolyte interface with additives in electrolytes to form a more stable SEI [14–16]. For instance, dense and insoluble components of Li_2_O and LiF were formed by the addition of a small amount of HF, which could improve the CE during cycling [16]. But the volume change upon cycling remains a problem. Another strategy involves optimizing the electrodes for minimizing the volume change and inhibiting the development of Li dendrites. To do this, conductive or insulating frameworks with three-dimensional (3D) structures have been used to provide larger surface areas and volumes [11, 17–20]. As the Sand's law indicates that the time needed for dendrite growth is inversely proportional to the square of the local current density and the anionic mobility [21], designing a high-surface-area anode composite is considered beneficial for the suppression of dendrite growth [17, 22]. However, these porous structures may reduce the packing density, leading to a lowered volumetric capacity incomparable to those densely-packed materials [23]. Introducing interlayer materials or modifying separators, on the other hand, also shows a potential to stabilize the Li metal. For example, glass fibers have been reported as the interlayer between the separator and Cu, in which the polar functional groups on the fibers may adsorb Li ions to compensate for the preferential deposition of Li on the rough Cu surface, thus leading to a more uniform Li deposition [20]. Similarly, a membrane consisting of submicron *α*-Si_3_N_4_ wires has been proposed as an interface to prolong the lifetime of Li metal batteries, in which the tortuous pores were thought to extend the physical path of growing dendrites and thus effectively suppress the dendrite growth towards the separator [24]. The nonelastic interface, however, still suffers from the loose interfacial contact and fluctuating stress during the repeated plating/stripping, which increases the interface instability [25].

Recently, a stress-driven dendrite growth model has been proposed to explain the drastic difference in Li growth behavior on hard versus soft substrates; the mitigation of dendrite growth was observed on soft substrates in electrodeposition [6]. Benefitting from flexibility, polymers are of intense interest as elastic interfaces on Li metal anodes [26–29]. For example, a porous polydimethylsiloxane (PDMS) film obtained by HF etching [29] and a flexible artificial interphase layer fabricated by a chemical reaction between the Li metal and polyacrylic acid [30] have resulted in a more stable interface and more uniform Li deposition than the unmodified electrodes. With such an elastic interface, the distribution of Li deposition could be regulated and the volume change minimized as these polymers can provide a conformal contact with the Li metal. In this sense, a nonchemical and scalable manufacturing of an elastic interlayer is valuable. In another report, an enhanced CE of 94.7% after 50 cycles of measurement at a current density of 10 mA cm^−2^ was achieved by placing a porous poly(melamine-formaldehyde) (PMF) on a Cu foil in a half cell (Li‖separator/PMF‖Cu), which has been attributed to homogenized Li distribution and reduced volume change in the PMF host [28]. In the measurements, however, prestoring Li (20 mA h cm^−2^) by electrodeposition in PMF is required before the cycling, which is time consuming and complicated in practical applications. In addition, cycling at the higher areal capacity (>5 mA h cm^−2^) is needed to verify the effect of such elastic interface for a higher capacity; the interfacial stability with such an interface remains insufficiently understood. Therefore, a strategy using a more practical preparation technique towards superior cycling ability under simultaneously high areal capacity and high current density is highly desired [6, 17].

In this work, an elastic yet porous interface for Li metal anodes is designed by a simple compression of the malleable Li metal with a melamine sponge (noted as MS-Li). With the good elasticity of the sponge, the obtained elastic interface enables a more sufficient interfacial contact during cycling and a more compact deposition of Li in plating, resulting in a CE of 98.8% for a high capacity of 10 mA h cm^−2^. Besides, it was found that the melamine sponge (noted as MS) can be applied independently as an elastic separator in symmetric cells to replace the conventional polypropylene (PP) separator, showing superior cycling and rate performance as well. The effect of the elastic interface on cycling stability is also confirmed by an in situ compression-electrochemistry measurement.

## 2. Results

**Figure 1:**
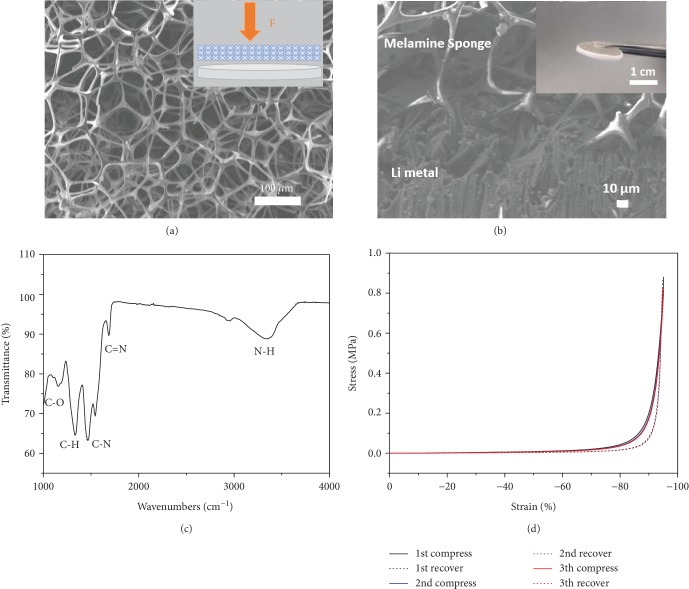
Schematic of the preparation and characterization of MS and MS-Li. (a) Typical SEM image of MS. Inset shows the schematic of the manufacturing method. (b) Cross-sectional SEM image of the MS-Li interface, showing the integrated structure of the composite anode. Inset shows an optical image of the MS-Li composite anode. (c) The FTIR spectrum of MS and (d) stress-strain curves of MS with compression strain varying from 0% to 95%.

The manufacturing procedure of the MS-Li composite anode is briefly illustrated in the inset of Figure 1(a), and the detailed experimental description is provided in Supporting Information. Briefly, the composite anode was obtained by the axial compression of a melamine sponge (MS) with a malleable Li foil. As shown in Figure 1(a), the scanning electron microscopy (SEM) image shows that MS has a three-dimensional (3D), cross-linked porous structure consisting of interconnected fibers. The pore size of MS is mainly distributed in the range of 50~100 *μ*m ([Supplementary-material supplementary-material-1], Supporting Information). After compression, the optical photograph (inset of Figure 1(b)) of the free-standing MS-Li composite shows that one side of the Li metal is uniformly covered by MS, and the compressed MS has a much smaller pore size due to the deformation of big pores ([Supplementary-material supplementary-material-1], Supporting Information). The SEM image in Figure 1(b) further shows that the 3D porous structure of MS is preserved, and the skeleton of MS penetrates into the surface of the Li foil (Figure 1(b)) due to the malleable nature of Li and the high elasticity of MS. The Fourier transform infrared (FTIR, Figure 1(c)) spectrum of MS shows vibration peaks corresponding to C-O at 1163 cm^−1^, C-H at 1342 cm^−1^, C-N at 1463 cm^−1^, C=N at 1690 cm^−1^, and N-H at 3347 cm^−1^ [31], respectively. X-ray photoelectron spectroscopy (XPS) measurement ([Supplementary-material supplementary-material-1], Supporting Information) indicates that MS is composed of N (27.57 at.%), C (58.09 at.%), and O (14.34 at.%). The XPS N1s spectrum ([Supplementary-material supplementary-material-1], Supporting Information) of MS can be deconvoluted into peaks of pyridinic-N (N-6, ~398.5 eV), pyrrolic-N (N-5, ~399.9 eV), and quaternary-N (Q-N, ~401.1 eV) [32], respectively. The XPS C1s spectrum ([Supplementary-material supplementary-material-1], Supporting Information) also shows the existence of C-O, which is consistent with FTIR. It has been reported that the polar groups (amine and ether) of MS have strong affinity with Li ions and may promote the uniform deposition of Li ions in plating [27, 28]. As can be seen from the mechanical compression testing in Figure 1(d), MS is elastic up to a compressive strain of 95% and the excellent consistency among curves indicates the potential ability of MS as an elastic interface to relieve strain upon cycling. [Supplementary-material supplementary-material-1] in Supporting Information shows that MS demonstrates a dimensional shrinkage much lower than the conventional polypropylene (PP) separator when both were heated at 150°C for 1 h, indicating the better thermal stability of MS.

**Figure 2:**
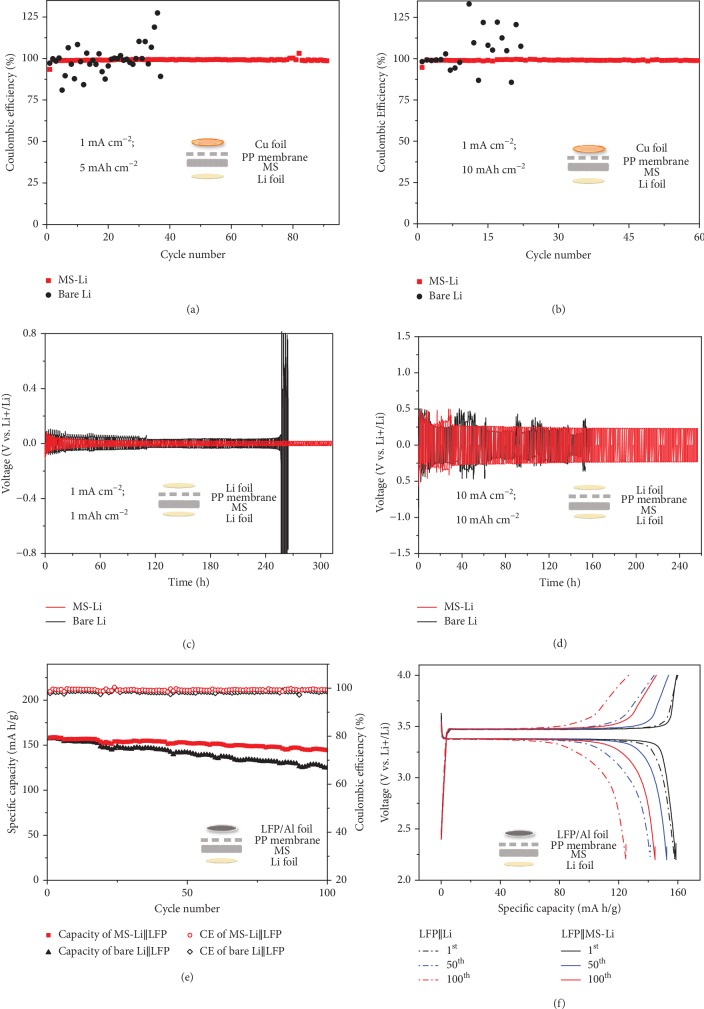
Electrochemical evaluation of cells with MS-Li or bare Li as anodes. Coulombic efficiencies of MS-Li and bare Li anodes measured at the current of 1 mA cm^−2^ for (a) a capacity of 5 mA h cm^−2^ and (b) a capacity of 10 mA h cm^−2^. Voltage profiles in the cycling of Li‖MS-Li and Li‖Li symmetric cells for (c) a capacity of 1 mA h cm^−2^ at a current of 1 mA cm^−2^ and (d) a capacity of 10 mA h cm^−2^ at a current of 10 mA cm^−2^. (e) Cycling performance of LFP‖MS-Li and LFP‖Li full cells at 0.5 C. (f) Charge and discharge voltage profiles of LFP‖MS-Li and LFP‖Li full cells of the 1^st^, 50^th^, and 100^th^ cycle. Insets show the corresponding structures of all the cells, respectively.

The Coulombic efficiency (CE) of MS-Li anodes has been systemically compared to that of bare Li via galvanostatic discharge/charge profiles of half cells with bare Cu as the cathode and PP as the separator in between. As shown in [Supplementary-material supplementary-material-1] in Supporting Information, the elastic MS-Li anode shows a CE of 98.5% for over 200 cycles at 1 mA cm^−2^ for an areal capacity of 1 mA h cm^−2^, while the bare Li anode shows a rapidly decaying and fluctuating CE after 53 cycles. When the areal capacity is increased to 3 mA h cm^−2^, the MS-Li anode exhibits a CE of 99.1% for over 170 cycles at 1 mA cm^−2^, but the bare Li shows large oscillation and faster fading after 10 cycles ([Supplementary-material supplementary-material-1]). Figures [Supplementary-material supplementary-material-1] and [Supplementary-material supplementary-material-1] further indicate that the MS-Li anode possesses a more stable stripping capacity upon cycles compared with the bare Li anode. More impressively, when the areal capacity is further increased to 5 mA h cm^−2^ (Figure 2(a)) or 10 mA h cm^−2^ (Figure 2(b)), the MS-Li anode still maintains a CE of 98.7% for 90 cycles or 98.8% for 60 cycles. In contrast, the bare Li anode displays fluctuating CE and increased instability for such high capacities, consistent with previous reports [33, 34]. It is worth noting that most CE measurements reported before were performed under areal capacities ≤ 5 mA h cm^−2^ [35, 36]. The CE cycling of the MS-Li anode is comparable or superior to previous reports in terms of areal capacity, current density, and CE lifetime ([Supplementary-material supplementary-material-1] in Supporting Information) [6, 17, 28, 33, 36–38].

To further evaluate the cycling performance, the voltage variation has been monitored during Li plating and stripping in symmetrical cells (configurations shown in insets).  Figure 2(c) shows the voltage profiles of MS-Li and bare Li anodes at 1 mA cm^−2^ for a capacity of 1 mA h cm^−2^, in which the MS-Li anode exhibits a very low overpotential of ~25 mV for 311 h, while the bare Li anode shows a larger overpotential of ~45 mV with fluctuations and a dramatic increase of voltage after 250 h, indicating the depletion of electrolytes or the failure of the electrode/electrolyte interface [36, 37]. For the simultaneously high current of 10 mA cm^−2^ and a high areal capacity of 10 mA h cm^−2^ (Figure 2(d)), the MS-Li anode still shows a stable overpotential of ~250 mV for 256 h, while the bare Li anode behaves like random oscillation and eventually experiences a sudden voltage drop after 138 h. Full cells were also fabricated by using the bare Li metal or MS-Li as anodes and commercial LiFePO_4_ as cathodes (denoted by LFP‖Li and LFP‖MS-Li, respectively) to explore the potential for practical applications. As shown in Figure 2(e), both full cells deliver similar initial discharge capacities and high initial CEs at 0.5 C (158 mA h g^−1^ with a CE of 98.7% for LFP‖MS-Li and 157 mA h g^−1^ with a CE of 97.9% for LFP‖Li). After 100 cycles, however, the capacity retention of LFP‖MS-Li is 91.1%, while that of LFP‖Li is only 79.2%. The voltage profiles shown in Figure 2(f) also support the superior cycling performance of LFP‖MS-Li as the LFP‖MS-Li demonstrates a smaller polarization of curves than LFP‖Li between the 1^st^ and 100^th^ cycles under the same conditions.

**Figure 3:**
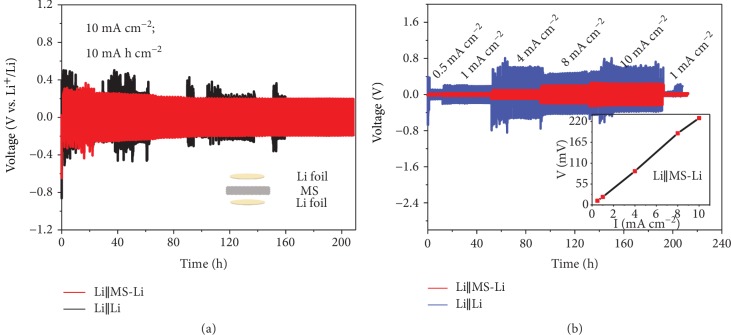
Electrochemical performance of using MS as an independent separator. (a) Voltage profiles of a symmetrical cell directly using MS as a separator (Li‖MS-Li), compared to a conventional cell with a PP separator (Li‖Li). Inset shows the structure of Li‖MS-Li. (b) Rate performances of two cells at currents from 0.5 to 10 mA cm^−2^. Inset shows the evolution of overpotential of Li‖MS-Li depending on the current.

Furthermore, we demonstrate that the porous and insulating MS can be used to replace the conventional PP separator; that is, a cell can be directly assembled by stacking Li and MS-Li together, as shown in the inset of Figure 3(a). The voltage profiles of Li‖MS-Li and Li‖Li at 10 mA cm^−2^ for 10 mA h cm^−2^ are compared in Figure 3(a). As we can see, Li‖MS-Li shows a low overpotential of 200 mV and a stable cycling for 208 h, while Li‖Li exhibits fluctuating overpotentials and eventually a short circuit after 138 h. Figure 3(b) shows the rate performances of the two cells evaluated by cycling the cells under current densities from 0.5 to 10 mA cm^−2^ for 1 h in each half cycle. For the current densities of 0.5, 1, 4, 8, and 10 mA cm^−2^, the overpotential of Li‖MS-Li is 11, 21, 89, 189, and 229 mV, respectively. As shown in the inset of Figure 3(b), the overpotential of Li‖MS-Li shows a nearly linear dependence on the current density and remains relatively low and stable during cycling compared to Li‖Li, which further demonstrates the effectiveness of MS-Li in LMBs. The stability for the elastic interface was further investigated by SEM before or after 10 mA h cm^−2^ of Li is stripped in a symmetric cell. Figures [Supplementary-material supplementary-material-1] and [Supplementary-material supplementary-material-1] in Supporting Information show that after 10 mA h cm^−2^ of Li (corresponding to a pure Li thickness of ~50 *μ*m) is stripped, MS is still attached to the Li metal with a part penetrating into the upper surface of Li and the thickness of residual Li in the MS-Li anode is reduced to ~410 *μ*m from the original ~460 *μ*m. That is, the self-adapting feature of the elastic interface helps to maintain the pressure on the Li metal upon cycling, resulting in less Li residue and higher cycling stability for a high areal capacity with MS on the Li metal [28, 39].

**Figure 4:**
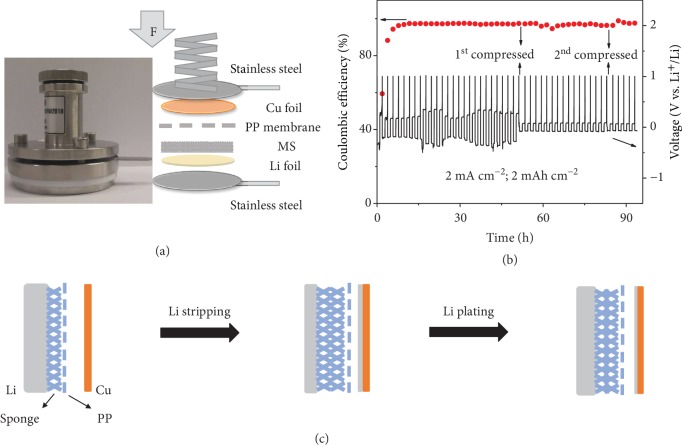
In situ compression-electrochemistry evaluation. (a) Optical photograph and schematic of the cell for applying pressure during cycling. (b) In situ compression-electrochemistry evaluation and voltage profiles of MS-Li at 2 mA cm^−2^ for 2 mA h cm^−2^. (c) Schematic showing the changes of a sponge-driven elastic interface in the process of Li stripping and plating.

To further understand the role of the MS elastic interface in the cycling performance, an in situ compression-electrochemistry testing was carried out at a current of 2 mA cm^−2^ and a capacity of 2 mA h cm^−2^.  Figure 4(a) shows the optical photograph and the schematic of the cell for electrochemical testing. With such a device, the pressure applied on the anodes could be changed via a spring by the screw on the top.  Figure 4(b) shows that the MS-Li anode could achieve a stable CE of 97.1% with an overpotential of ~270 mV for 25 cycles under the initial state (no obvious deformation of the spring). When the axial pressure is increased by another ~0.2 MPa (estimated by the deformation degree of the spring, which is calibrated with a known weight), the CE remains at 96.4% with a much lower overpotential of 85.6 mV till the 43^rd^ cycle, when axial pressure is further increased by ~0.4 MPa. After that, the MS-Li anode maintains a CE of 97.5% with an overpotential of 68.2 mV till the end of the measurement. The lower overpotential under higher axial pressures indicates the better electrical contact and more compact Li deposition under more compression of the elastic interface [34, 39]. The effect of compression has also been confirmed by the cycling of a symmetrical cell using a thinner MS while keeping all other components the same. As shown in [Supplementary-material supplementary-material-1] (Supporting Information), the cell with 1 mm MS delivers the higher overpotential and shorter cycling, compared to the cell with 2.5 mm MS (Figure 1(d)) under the same measurement conditions. This can be explained by the effect that the thinner MS would have the larger pore size and smaller strain under the same compression force in the assembled cell. The role of the elastic interface upon cycling is more clearly illustrated by the schematic in Figure 4(c). In the process of Li stripping, the elastic interface could enable more sufficient interfacial contact and reduce the formation of isolated Li [30, 40]. In the Li plating process, the gradually increased thickness of the Li layer leads to the higher compression of the sponge and more compact deposition of Li. Thus, the 3D porous elastic interface performs as a self-adapting layer to provide a suitable pressure and alleviate the volume change by forming a dense and uniform Li during the dynamic process of charging and discharging [39, 40].

**Figure 5:**
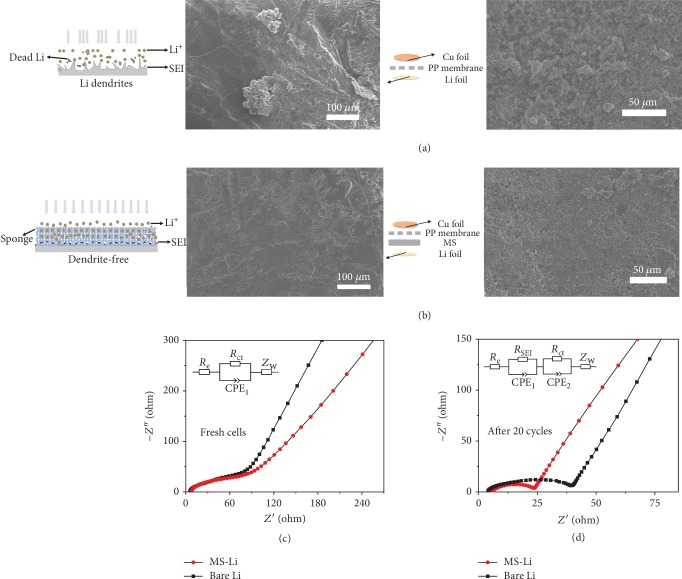
Characterizations of the interface in MS-Li and bare Li anodes. Schematic illustration of electrochemical deposition and SEM images of (a) the bare Li anode and (b) the MS-Li anode after 30 cycles. The electrodes were taken from half cells with a cycling capacity of 1 mA h cm^−2^ and a current density of 1 mA cm^−2^. The surface morphology of the corresponding Cu foils has also been shown, respectively. Nyquist plots of half cells using MS-Li and bare Li anodes measured at (c) a fresh state and (d) after 20 cycles under the capacity of 1 mA h cm^−2^ and current of 1 mA cm^−2^. Insets show the corresponding fitting model.

The morphological investigation for MS-Li and bare Li anodes was performed by disassembling the half cells after 30 cycles carried out at the current of 1 mA cm^−2^ for the capacity of 1 mA h cm^−2^. As can be seen from Figure 5(a), an obvious tendency to pulverization is observed on bare Li, which could further accelerate the heterogeneous plating and stripping of Li ions in return [13, 41]. Meanwhile, the rough morphology of deposited Li with numerous protrusions on the surface of Cu is also observed from Figure 5(a), indicating the nonuniform deposition of Li and the loose contact between Li particles on Cu in bare Li‖Cu [28, 42]. In contrast, Figure 5(b) shows a relatively more compact and smoother surface of Li in MS-Li (after MS was peeled off), suggesting that the elastic interface favors the more homogeneous distribution of Li ions and also provides a suitable pressure to suppress the volume change during the dynamic process, eventually forming an even and dense Li layer [13]. The measurement performed at a higher current density and higher capacity (e.g., 5 mA cm^−2^ and 5 mA h cm^−2^) also results in the similar observation ([Supplementary-material supplementary-material-1], Supporting Information). Moreover, a compact and dendrite-free morphology on the Cu foil has also been achieved with the elastic MS-Li anode, which further demonstrates the role of regulating the deposition of Li ions by MS in MS-Li‖Cu. Electrochemical impedance spectroscopy (EIS) of half cells using MS-Li or the bare Li anode (Figures 5(c) and 5(d)) was performed to evaluate the ion transportation and interfacial stability before and after cycling, as shown in Figure 5(c). The semicircle at the high frequency range in Nyquist plots indicates the interfacial resistance and that at the low frequency range indicates the ion diffusion-limited process contributing to the Warburg impedance *Z*_W_ response [22, 43]. By fitting the EIS curves, the resistances were estimated and listed in [Supplementary-material supplementary-material-1] (Supporting Information). As can be seen from Figure 5(c), the MS-Li and bare Li anodes display a similar interfacial resistance (~100 Ω) under a fresh state. After 20 cycles were performed with a capacity of 1 mA h cm^−2^ and a current of 1 mA cm^−2^, however, the MS-Li anode displays a much lower interfacial resistance of 22.7 Ω, compared to 38.9 Ω for the bare Li anode. The performance of the MS-Li anode is also comparable to the previous reports on interface engineering [11, 28], suggesting that the stable interface and better contact can be achieved by the elastic MS.

## 3. Discussion

In summary, we have fabricated an elastic interface on the Li anode to stabilize the reaction by inserting a melamine sponge between Li and the separator; superior CE and cycling stability have been achieved at a high current density of 10 mA cm^−2^ and a high capacity of 10 mA h cm^−2^. The outstanding performance has been attributed to the elastic interface since more sufficient interfacial contact during Li plating and stripping shall prevent the formation of isolated Li and fractioned SEI. A homogeneous distribution of Li ions has also been obtained due to the regulation of the porous structure and the polar groups of the melamine sponge, resulting in a compact and smooth Li layer stable for long cycles. This simple, facile, yet effective method may shed light on further research on Li metal anodes by rationally designing self-adapting elastic interfaces.

## 4. Materials and Methods

### 4.1. Materials and Preparation of MS-Li

In a typical procedure, a piece of as-purchased melamine sponge (Puyang En-World New Material Co. Ltd., China) was cut into slices with a relaxed thickness of 2.5 mm and punched into round electrodes with a diameter of 16 mm and a mass of 5 mg. Then, the sponge was compressed with a Li metal (Wuhan Newthree Technology Co. Ltd., China) with a diameter of 14 mm by axial compression for the MS-Li composite. Based on the exponential fitting of the stress-strain curves, the thickness of MS was estimated as ~25 *μ*m, comparable to that of commercial PP (~24 *μ*m).

### 4.2. Structural Characterizations

The morphology of the samples was characterized by scanning electron microscopy (SEM) (JEOL JSM-6700F and FEI Sirion 200). The composition of samples was evaluated with Fourier transform infrared spectroscopy (FTIR, Nicolet 8700) and X-ray photoelectron spectroscopy (XPS, ESCALAB 250). The mechanical property of the samples was measured by a compression test (Instron 5565A) with the compressive strain from 0 to 95% in each cycle.

### 4.3. Electrochemical Measurements

Coin batteries (CR2032 type) were assembled for all electrochemical measurements in an argon-filled glove box (Vacuum Technology Inc., USA), in which H_2_O and O_2_ contents were below 0.1 and 0.1 ppm, respectively. 1 M lithium bis(trifluoromethanesulfonyl)imide in 1,3-dioxolane (DOL)/1,2-dimethoxyethane (DME) (1 : 1 *w*/*w*) with 2 wt.% lithium nitrate was used as an electrolyte, as a previous study has shown that it can protect the Li metal anode and enable the long-term cycling stability [44]. A porous polypropylene membrane (Celgard 2400) served as a separator in a conventional structure. Typically, ~105.75 mg electrolyte was used in MS-Li cells. The electrochemical measurements were executed using a LAND multichannel battery test system (Wuhan, China). Electrochemical impedance spectroscopy (EIS) measurements were performed on a PARSTAT 4000 electrochemical workstation (USA) with a voltage amplitude of 5 mV and a frequency range of 10^−1^-10^5^ Hz. To evaluate the Coulombic efficiency (CE), a certain amount of Li (1, 3, 5, and 10 mA h cm^−2^) was deposited onto a Cu substrate and then charged to 1.0 V (versus Li^+^/Li) to strip the Li at the same current density of 1 mA cm^−2^ in each cycle. The CE was calculated based on the ratio of the amount of stripping Li versus the amount of depositing Li [13]. Full batteries were assembled with MS-Li or bare Li metal as anodes and using LiFePO_4_ (was purchased from Suzhou Aimeide New Energy Material Co. Ltd., China) as cathodes, both under the same experimental conditions. The cathodes were made by LiFePO_4_, acetylene black, and polyvinylidene fluoride (PVDF) (8 : 1 : 1), which were mixed in N-methyl-2-pyrrolidone (NMP) to form a homogeneous slurry and pasted on an Al foil, then dried at 80°C under vacuum overnight. The mass loading of LFP is about 2.5 mg cm^−2^. All the full cells were cycled in a galvanostatic mode with a voltage range of 2.2 to 4.0 V.

## Supplementary Materials

Supplementary Materials Figure S1: typical SEM images of (a) relaxed and (b) compressed MS. Figure S2: (a) elemental analysis of MS and high-resolution XPS spectra of (b) N1s and (c) C1s with deconvolution. Figure S3: thermal stability test of MS and conventional polypropylene membrane at 150°C for 1 h. Figure S4: Coulombic efficiency evaluation of MS-Li and bare Li anodes measured at the current density of 1 mA cm^−2^ for (a) the areal capacity of 1 mA h cm^−2^ and (b) the areal capacity of 3 mA h cm^−2^. Voltage profiles of Li plating/stripping on (c) the MS-Li anode and (d) the bare Li anode in different cycles with a capacity of 3 mA h cm^−2^ at a current of 1 mA cm^−2^. Figure S5: cross-sectional SEM images of (a) a fresh Li anode before being paired with MS and (b) the MS-Li anode after stripping Li for a capacity of 10 mA h cm^−2^. Figure S6: cycling of MS with a lower compression ratio due to the thinner MS used in a symmetrical cell. Figure S7: SEM images of (a) the bare Li and (b) the MS-Li anode after 30 cycles performed at 5 mA cm^−2^ and for 5 mA h cm^−2^. Table S1: comparison with recent literatures regarding lithium metal anodes in the CE test. Table S2: EIS fitting parameters of half cells before and after cycling.
