# Supplementary material for: A Sponge-Driven Elastic Interface for Lithium Metal Anodes
Source: Research (Wash D C). 2019 Sep 15;2019:9129457. doi: 10.34133/2019/9129457 (PMC6946253; doi:10.34133/2019/9129457)
Supplement: Supplementary Materials — Figure S1: typical SEM images of (a) relaxed and (b) compressed MS. Figure S2: (a) elemental analysis of MS and high-resolution XPS spectra of (b) N1s and (c) C1s with deconvolution. Figure S3: thermal stability test of MS and conventional polypropylene membrane at 150°C for 1 h. Figure S4: Coulombic efficiency evaluation of MS-Li and bare Li anodes measured at the current density of 1 mA cm−2 for (a) the areal capacity of 1 mA h cm−2 and (b) the areal capacity of 3 mA h cm−2. Voltage profiles of Li plating/stripping on (c) the MS-Li anode and (d) the bare Li anode in different cycles with a capacity of 3 mA h cm−2 at a current of 1 mA cm−2. Figure S5: cross-sectional SEM images of (a) a fresh Li anode before being paired with MS and (b) the MS-Li anode after stripping Li for a capacity of 10 mA h cm−2. Figure S6: cycling of MS with a lower compression ratio due to the thinner MS used in a symmetrical cell. Figure S7: SEM images of (a) the bare Li and (b) the MS-Li anode after 30 cycles performed at 5 mA cm−2 and for 5 mA h cm−2. Table S1: comparison with recent literatures regarding lithium metal anodes in the CE test. Table S2: EIS fitting parameters of half cells before and after cycling. [file 9129457.f1.docx]

Supporting Information

**A Sponge-Driven Elastic Interface for Lithium Metal Anodes**

Han Yu^1,^ **^†^**, Jian Xie^1,^ **^†^**, Na Shu^1^, Fei Pan^1^, Jianglin Ye^1^, Xinyuan Wang^1^, Hong Yuan^1^, and Yanwu Zhu^1, 2,^ *

^1^ Hefei National Research Center for Physical Sciences at the Microscale, & Department of Materials Science and Engineering, & CAS Key Laboratory of Materials for Energy Conversion, University of Science and Technology of China, Hefei, Anhui 230026, P. R. China

^2^ iChEM (Collaborative Innovation Center of Chemistry for Energy Materials), University of Science and Technology of China, Hefei, Anhui 230026, P. R. China.

^†^ These authors contributed equally to this work.

* Correspondence should be addressed to Yanwu Zhu; [zhuyanwu@ustc.edu.cn](mailto:zhuyanwu@ustc.edu.cn)


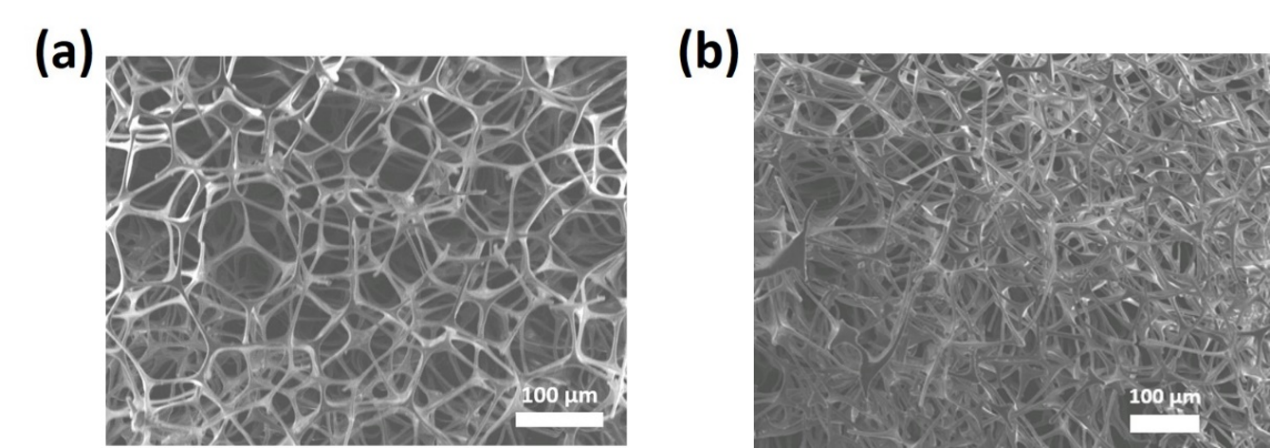
Figures

Figure S1: Typical SEM images of (a) relaxed and (b) compressed MS.


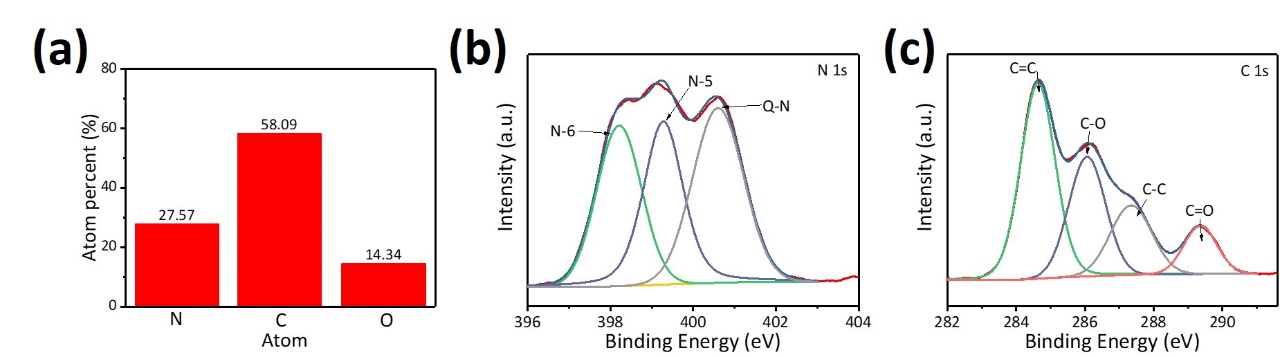


Figure S2: (a) Elemental analysis of MS, and High-resolution XPS spectra of (b) N1s and (c) C1s with deconvolution.


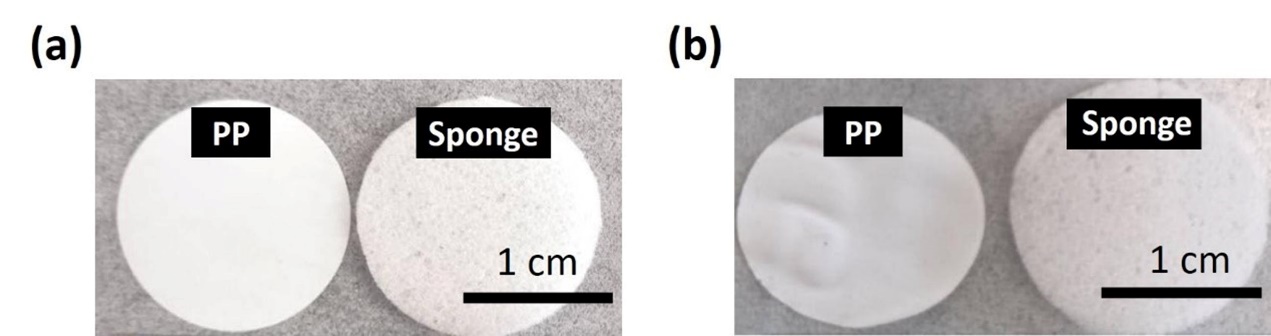


Figure S3: (a) Thermal stability test of MS and conventional polypropylene (PP) membrane (a) under initial state and (b) after being heated at 150℃ for 1 h.


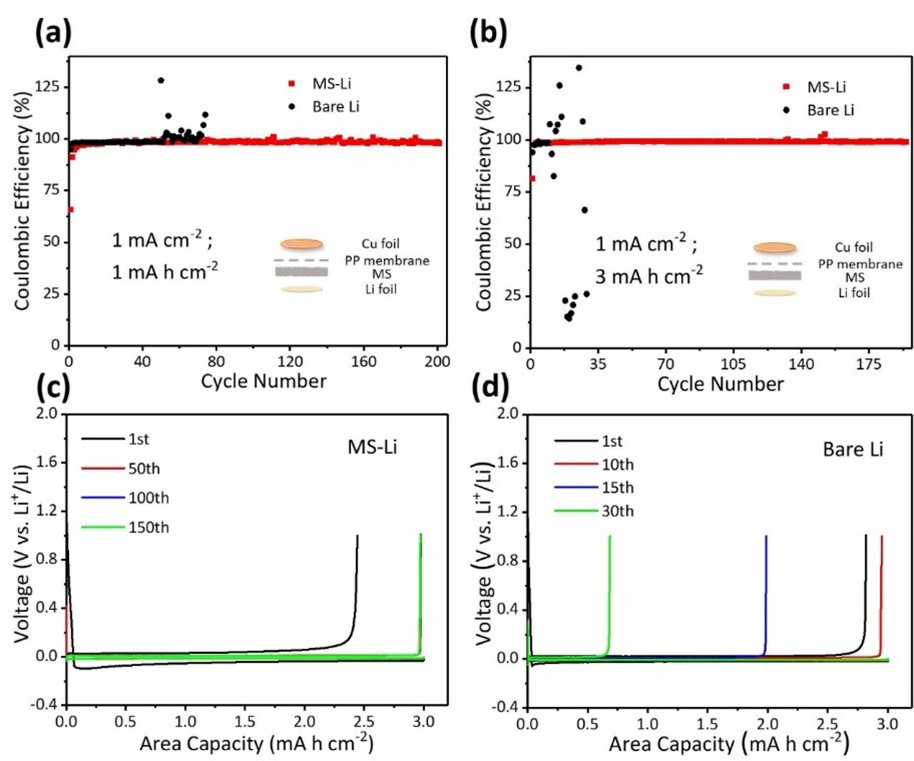


Figure S4: Coulombic efficiency evaluation of MS-Li and bare Li anodes measured at the current density of 1 mA cm^-2^ for (a) areal capacity of 1 mA h cm^-2^ and (b) areal capacity of 3 mA h cm^-2^. Voltage profiles of Li plating/stripping on (c) MS-Li anode and (d) bare Li anode in different cycles with a capacity of 3 mA h cm^-2^ at current of 1 mA cm^-2^.


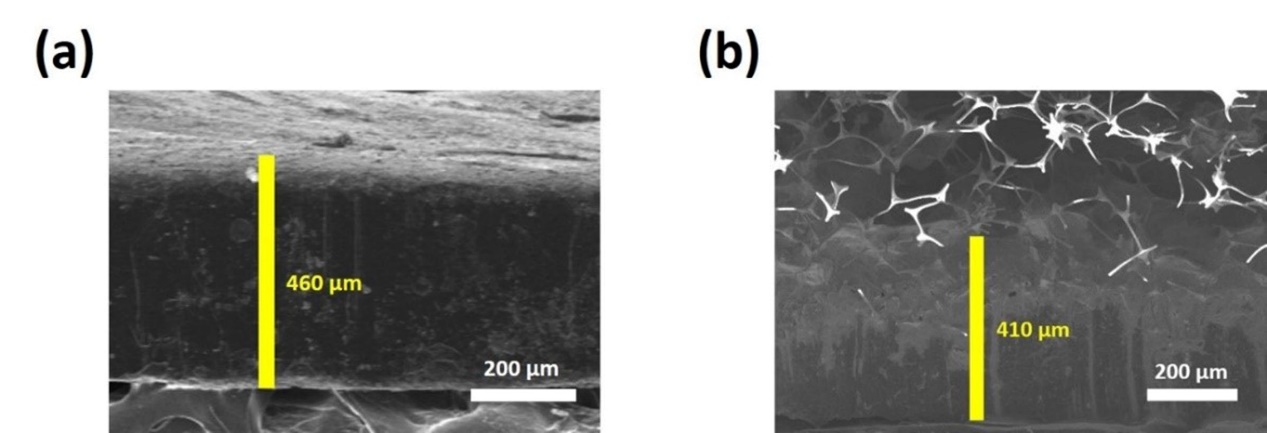


Figure S5: Cross-sectional SEM images of (a) fresh Li anode before being paired with MS and (b) MS-Li anode after stripping Li for a capacity of 10 mA h cm^-2^.


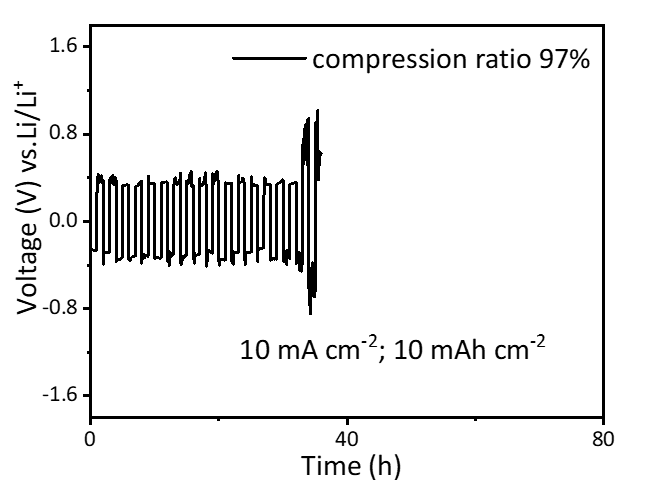


Figure S6: Cycling of MS with lower compression ratio due to the thinner MS used in a symmetrical cell.


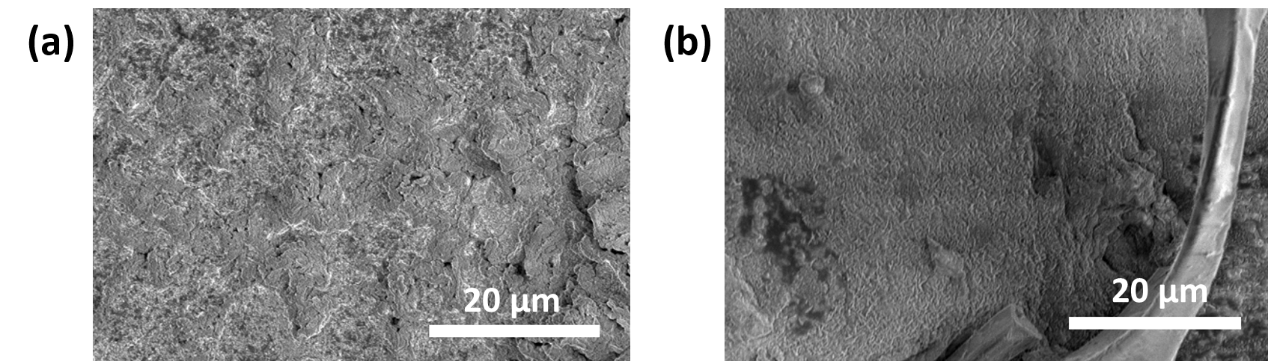


**Figure S7:** SEM images of (a) bare Li and (b) MS-Li anode after 30 cycles performed at 5 mA cm^-2^ and for 5 mA h cm^-2^.

Tables

**Table S1:** Comparison with recent literatures regarding lithium metal anodes in CE tests. The areal capacity during cycles (including the cycling numbers at each condition) and current density are taken into consideration.

| Approaches and references | Areal capacity during cycling (mA h cm^-2^) /n(cycles) | Current density (mA cm^-2^) |
| --- | --- | --- |
| Stress-driven Li growth [6]  GT-scaffold [18]  Sponge host [29]  Crumpled graphene balls [34] | 3/75  1/200  10/100  1/200  1/140  1/50  2/188 | 3  1  5  1  3  10  0.5 |
| Flexible-grid composite [37] | 2/250  1/20 | 2  1 |
| Cu foam as current collector [38] | 1/150  1/250 | 2  0.5 |
| N-doped graphene matrix [39] | 1/200  2/50 | 1  1 |
| **This work** | **1/201**  **3/194**  **5/91**  **10/60** | **1**  **1**  **1**  **1** |

**Table S2:** EIS fitting parameters of half cells before and after cycling.

|  | Fresh cell (Ω) | | | After 20 cycles (Ω) | | | |
| --- | --- | --- | --- | --- | --- | --- | --- |
|  | R_Ω_ | R_interface_ | R_total_ | R_Ω_ | R_SEI_ | R_ct_ | R_total_ |
| MS-Li | 4.7 | 100.2 | 104.9 | 3.9 | 15.8 | 3.0 | 22.7 |
| Bare Li | 3.5 | 99.6 | 103.1 | 3.8 | 27.9 | 7.2 | 38.9 |
